# How efficient are German life sciences? Econometric evidence from a latent class stochastic output distance model

**DOI:** 10.1371/journal.pone.0247437

**Published:** 2021-03-12

**Authors:** Denitsa Angelova, Maya Göser, Stefan Wimmer, Johannes Sauer

**Affiliations:** Chair Agricultural Production and Natural Resource Economics, Technical University of Munich, Munich, Bavaria, Germany; Universitá Cattolica del Sacro Cuore, ITALY

## Abstract

This article investigates the technical efficiency in German higher education while accounting for possible heterogeneity in the production technology. We investigate whether a latent class model would identify the different sub-disciplines of life sciences in a sample of biology and agricultural units based on technological differences. We fit a latent class stochastic frontier model to estimate the parameters of an output distance function formulation of the production technology to investigate if a technological separation is meaningful along sub-disciplinary lines. We apply bootstrapping techniques for model validation. Our analysis relies on evaluating a unique dataset that matches information on higher educational institutions provided by the Federal Statistical Office of Germany with the bibliometric information extracted from the ISI Web of Science Database. The estimates indicate that neglecting to account for the possible existence of latent classes leads to a biased perception of efficiency. A classification into a research-focused and teaching-focused decision-making unit improves model fit compared to the pooled stochastic frontier model. Additionally, research-focused units have a higher median technical efficiency than teaching-focused units. As the research focus is more prevalent in the biology subsample an analysis not considering the potential existence of latent classes might misleadingly give the appearance of a higher mean efficiency of biology. In fact, we find no evidence of a difference in the mean technical efficiencies for German agricultural sciences and biology using the latent class model.

## Introduction

When resources are scarce it is vital that they are used efficiently. If public resources are concerned, it is in the governments’ interest to assure the efficient use of their invested means. This is especially true in the higher education sector as illustrated by the existence of the German University Excellence Initiative, which aims at the efficient distribution of public funds based on objective measures of academic performance. Similar efficiency considerations apply to the disciplinary context.

It is still the common practice at the level of a discipline to assume the same technology when estimating efficiency, though due to differing requirements within the disciplines it seems unlikely for them to behave in the same way and to share a common production technology and cost structure. German life sciences pose a particularly curious case in terms of technical efficiency quantification as they accommodate both biology departments and agricultural and nutritional science departments, which might exhibit an entirely different production logic.

In this study, we examine the technical efficiency of German life sciences while accounting for possible technological differences. In order to not a priori impose an assumption that these technological differences exist along sub-disciplinary lines we use a latent class model, which provides a data-driven method to endogenously classify the investigated units. We connect this latent class model to a stochastic frontier to estimate the parameters of an output-distance function formulation of the production technology at the level of a subject and research area (German: Lehr- und Forschungsbereich). Our research question thus relates to whether the latent class model would identify the different sub-disciplines of life sciences in a sample of biology and agricultural and nutritional science units based on technological differences. The terms “agricultural and nutritional sciences”, “agriculture” and “agricultural sciences” are used interchangeably henceforth.

This paper is organized as follows. Section 2 reviews the existing literature on efficiency in the context of academic research and higher education with a focus on the choice of the decision-making unit and the orientation of the production frontier (input vs. output-oriented). Section 3 describes the conceptual framework and proposes a model of scientific production, while section 4 outlines the data and formulates the empirical specification corresponding to the model. Section 5 presents the estimation results and offers a discussion. Section 6 provides a conclusion and an outlook.

## Literature review

Since the first introduction of efficiency models in the educational sector, not only the application of efficiency models has increased, but also the underlying methodologies have evolved. The paper by Charnes et al. was one of the first to link the methodology to the need of improving the planning and control of activities of decision-making units in public programs, schools in particular [1]. Soon, others recognized the use of efficiency calculation for higher education institutions and many coherences have been considered on different levels of analysis such as country level, university level or department level. Among the first to apply efficiency analysis in the higher education sector where Johnes und Johnes who in 1995 conducted a data envelopment analysis on cross-sectional data of 36 British university departments of economics [2]. Still, most research in this field has been conducted for the Anglo-Saxon area where mostly cross-sectional data is applied due to the legal and constitutional frameworks in UK higher education [3, 4]. The study of Johnes and Johnes from 2016 recognizes the diversity in the higher education sector and combines a latent class stochastic analysis with a cost frontier estimation [4]. This allows them to investigate the importance of heterogeneity on allocative efficiency. Their work builds on the random parameter cost approach to address heterogeneity adopted by Agasisti and Johnes (2010) in evaluating the allocative efficiency in the Italian case [5]. In a recent study from 2020 Wohlrabe and Gralka acknowledge the potential for heterogeneity of institutions and faculties of economics and classify them via archetypoid analysis [6]. They do not, however, investigate the implications of heterogeneity for the technical or allocative efficiency of the units they investigate.

Most studies focus on country-specific evaluations, but with rising data availability, cross-country studies that compare efficiency on the university level in different countries have become more popular. Public higher education institutions were studied using panel data at the European level using bootstrapped data envelopment models [7, 8]. Agasisti und Haelermans analyzed Italian and Dutch universities applying a stochastic frontier analysis on panel data covering the years 2005–2009 [9]. Agasisti and Gralka compare Italian and German universities [10]. All cross-country analyses have been conducted on the university level.

A literature review from the year 2015 by Witte and López-Torres suggests that the most common decision-making unit considered is a university and Rhaiem concurs in his study from 2017 [3, 11]. He also finds that the majority of academic articles examining the efficiency of research units are output-oriented. It is hypothesized that this is due to the specifics of academic production, namely maximizing outputs given a specific input level, rather than minimizing input commitment to maintain a certain level of output [3]. The majority of investigated studies adopts a non-parametric data envelopment approach to the analysis efficiency [3]. In her systematic review on stochastic frontier applications in higher education studies from the year 2018, Gralka identifies the cost function as the most used function to represent the technology, which implies an input orientation [12]. However, she acknowledges the rising importance of a primary distance function formulation in applied research.

An investigation of the literature regarding efficiency in German tertiary education shows a similar trend as described by Rhaiem in 2017: the majority of studies are output-oriented and investigate universities as a decision-making unit [13–29]. Parametric and non-parametric methods are used to a similar extent to investigate the German case. Since techniques like data envelopment and stochastic frontier where developed they have evolved substantially and gained popularity in many fields. Though accounting for heterogeneity is a widely discussed topic in the application of efficiency measurements [30], it has not yet found much attention when analyzing the German higher education sector. Although Grawellek und Sunder address the heterogeneity of universities with regard to their departments, they do not consider it in their empirical application [28]. While Pohl und Kempkes include a binary variable accounting for faculty composition to account for heterogeneity in their stochastic frontier analysis evaluating efficiency on the university level, they still assume a common shape of the production function for all decision-making units [15]. The effect of heterogeneity between German academic disciplines and sub-disciplines on efficiency scores has not yet been explored. In other sectors, it has already been recognized that falsely assuming a homogeneous technology can lead to biased estimation results. As Sauer has demonstrated, a latent class approach is applicable when heterogeneous technologies are observed and can produce a separate production frontier estimates for each latent class [31].

## A model of scientific production

We hypothesize that the production technology can be represented by a multiple-output, multiple-input distance function. This technology representation is not an uncommon choice, as the literature review by Gralka testifies, and would allow us to explicitly accommodate multiple outputs [12]. This is equivalent to effectively assuming that the decision-making unit can increase technical efficiency by increasing output, holding input levels constant. Functionally, the output distance function can be expressed as

$$D_{0}\left(x,y\right)=\min\;\left\{\theta :\;\frac{y}{\theta }\in Y\left(X\right)\right\}$$ (1)

An output distance function is by definition linearly homogeneous in outputs. It is thus true that for a positive *λ*:

$$D_{0}\left(x,\lambda y\right)=\lambda \;D_{0}\;\left(x,\frac{y}{\lambda }\right)$$ (2)

This theoretical property of the distance function facilitates the estimation of empirical specifications in the multiple-output setting. If the positive *λ* in (2) is substituted for one of the outputs that by definition takes on only positive values, say *y*_1_, then the flexible translog approximation of the multi-output production technology would be:

$$\begin{array}{l} \;-\ln\left(y_{1}\right)=\beta_{0}+\sum_{m=2}^{M}\beta_{m}\ln\left(\frac{y_{m}}{y_{1}}\right)+\sum_{n=1}^{N}\beta_{n}\ln x_{n}+0,5\sum_{m=2}^{M}\sum_{k=1}^{M}\beta_{y_{m}y_{k}}\ln\left(\frac{y_{m}}{y_{1}}\right)\ln\left(\frac{y_{k}}{y_{1}}\right)\\ +0,5\sum_{n=1}^{N}\sum_{k=1}^{N}\beta_{x_{n}x_{k}}\ln x_{n}\ln x_{k}+\sum_{m=2}^{M}\sum_{n=1}^{N}\beta_{y_{m}x_{n}}\ln\left(\frac{y_{m}}{y_{1}}\right)\ln x_{n}-\ln(D_{0}\left(x,\frac{y}{y_{1}}\right)) \end{array}$$ (3)

The variables in our model are selected based on the opinion of expert consultants in the structures of German higher education and on data availability consideration. We hypothesize that peer-reviewed papers, third party funding, undergraduate, graduate and PhD qualifications are produced by combining technical and scientific staff, undergraduate and graduate students as intermediate inputs for the qualifications and its scientific reputation as indicated by the citations of own articles accumulating in the course of the past five years.

The accommodation of peer-reviewed publications and research grants as outputs is also supported by Rheim [3] who identifies them as essential indicators for the academic performance. We regard the graduates as an output and the number of undergraduate and graduate students as an input similar to the approach adopted by Flegg et al [32]. The time-lag between immatriculation and graduation could not be considered since the student numbers represent averages between study programs with different duration. Due to the lack of a reliable statistic on the manner the number of doctoral candidates is merely reflected to the extent to which they belong to the scientific staff, which together with the technical staff constitutes the labor input [33]. Capital input to production is captured by the material costs (German: Sachkosten), which includes the capital depreciations, but also energy and material costs, rents and related services [34].

We understand citation numbers as an expression of scientific reputation, thereby an input which allows the decision-making units to attract students, scientific collaborators and research grants rather. This interpretation is supported by Rheim who notes that the ability of universities to appeal to international students depends on the reputation of their research [3]. Abramo et al [35] show a connection between scientific meritocracy as measured by the ratio of citations to publications and the intensity of university-industry research collaboration, which also speaks in favour of the accommodation of citations as an input. We disregard possible heterogeneity in the citation behaviour between the sub-disciplines of life sciences because agricultural sciences accommodate many biotechnology departments. A five year window has been chosen for the citation numbers to match the approximate duration of doctoral degrees, which are accommodated as outputs.

The decision-making unit (hereafter referred to as unit) is a German life science subject and research area producing research and teaching with capital, labour and intermediate expenditures. A subject and research area (German: Lehr- und Forschungsbereich) is a statistical unit used by the German Federal Office of Statistics (Destatis) to record the personnel numbers in higher education institutions [36]. The various outputs and inputs are listed in Tables 1 and 2 respectively. The subscripts *j* for unit and *t* for time are omitted for the sake of improved readability.

**Table 1:** Dimensions of output and the variables used to operationalize the dimensions.

Output category	Output	Denoted
Research	Number of publications	*y*_1_
Research	Third party funding procured	*y*_2_
Teaching	Number of undergraduate qualifications	*y*_3_
Teaching	Number of graduate qualifications	*y*_4_
Teaching	Number of PhD qualifications	*y*_5_

**Table 2:** Dimensions of input and the variables used to operationalize the dimensions.

Input category	Input	Denoted
Labor	Size of the technical staff	*x*_1_
Labor	Size of the scientific staff	*x*_2_
Intermediate Inputs	Number of undergraduate students	*x*_3_
Intermediate Inputs	Number of graduate students	*x*_4_
Intermediate Inputs	Number of citations (past five years)	*x*_5_
Capital	Material costs	*x*_6_

## Data and empirical specification

Our empirical analysis draws on a dataset documenting the period between 2005 and 2016 for 58 life science units in the 48 German universities listed in the Appendix I, which amounts to 696 observations. Ten of the 58 units belong to the agricultural science branch of life sciences, while the rest belong to the biological sciences branch. A selection of units was made from all possible units we could consider based on the focus of the study programs as German biology units also educate future high-school biology teachers. Units with a nearly exclusive focus on educating future teachers were omitted from the sample as they exhibit a different production logic than the rest.

The data on the selected units covers financial, personnel, examination and bibliometric aspects on the level of a subject and research area. The financial, personnel, examination data on the unit level is collected by the Federal Statistical Office of Germany (German: Statistisches Bundesamt) on an annual basis and made accessible via the ICEland platform after a rounding procedure (ICEland, 2020) [37]. The bibliometric data in the dataset originates from Web of Science and was accessed via the data infrastructure of the Competence Centre for Bibliometrics KZB, which maintains an in-house infrastructure to aid bibliometric applications (Competence Centre for Bibliometrics, 2020) [38]. The infrastructure has no standardisation of bibliometric data from Web of Science at the level of a subject and research area and the address lines on publications and key words proved unreliable for the identification of relevant publications. The topic classifications of the Web of Science are known to produce vastly inaccurate results in the case of agricultural sciences [39]. Therefore, the publication and citation data at the German subject and research area level were compiled using the following three-step procedure:

1. identifying the relevant subject and research area members via public records
2. extracting their individual publication and citation records from the database
3. summing up the publication and citation numbers to the subject and research area level at a university

We identify the names of the subject and research area members through public records (Hochschullehrer Verzeichnis 2007) [40]. The bibliometric dataset based on these three steps summarises the individual publication and citation records of approximately 5000 German life science professors, around 500 professors of agricultural sciences and around 4500 professors of biology. The untenured subject and research area members presumably tend to publish with tenured subject and research area members so that the obtained numbers are in general reliable. Random checks confirm the reasonability of this assumption.

Descriptive statistics on the mean, standard deviation, minimum and maximum values for the pooled sample (i.e. agricultural and biology) are presented in Table 3. There are two potential sources of zero values in the variables obtained from the ICEland database. On the one hand, the ICEland database uses a rounding procedure to protect personal data, which means that a value of zero could in actuality correspond to values of 0, 1 or 2 [41]. Many of the minima in the dataset were observed at the University of Wuppertal, where the relevant decision-making unit which belongs to the biology subsample recorded an average size of the scientific and technical staff of approximately 23. Values close to zero for at least one of the years seem plausible. On the other hand, both agricultural science and biology data display values consistent with the Bologna reform, i.e. a transition from the German Diplom degree, a single graduate degree, to a Bachelor/Master system means that some universities will have zero undergraduate qualifications and graduate students on record. The maxima with respect to student and qualification numbers are accounted for by the University of Hohenheim. A binary variable that accounts for the sub-discipline the specific decision-making unit belongs to completes the dataset.

**Table 3:** Descriptive statistics for the pooled sample before imputation.

Variable	Origin	Mean	Std.Dev.	Min	Max
Publications (Publ)	KZB	65.03	59.24	0	576
Third party funding (F*100k)	ICEland	12,612,852	10,082,318	0	62,927,879
Undergraduate qualifications (UG)	ICEland	64.95	61.07	0	400
Graduate qualifications (GrQ)	ICEland	88.33	54.59	0	475
PhD qualifications (PHDQ)	ICEland	48.73	36.52	0	250
Material costs (MC*100k)	ICEland	1,133,729	2,274,766	346	13,684,143
Technical staff (TS)	ICEland	73.74	51.44	0	400
Scientific staff (ScS)	ICEland	198.66	103.23	5	610
Undergraduate students (US)	ICEland	144.63	70.39	0	520
Graduate students (GS)	ICEland	56.92	60.17	0	465
Citations past five years (Cit*10)	KZB	1093.39	1270.8	1	7,187

Source: own calculation.

Tables 4 and 5 show the mean, standard deviation, minimum and maximum values for the agricultural sciences and biology subsamples respectively. The average agricultural science unit tends to have more graduate and undergraduate students, which naturally results in more graduate and undergraduate qualifications. Biology units tend to graduate more PhDs on average. Both are comparable in terms of third-party funding obtained from competitive grant submissions and publication counts, but biology publications are cited significantly more often. For both sub-disciplines the mean sum of undergraduates and graduates is close to the mean of the scientific staff, which should interpreted against the background that only few PhD students teach.

**Table 4:** Descriptive statistics for the agricultural science sample before imputation.

Variable	Mean	Std.Dev.	Minimum	Maximum
Publications	72.94	82.54	1	576
Third party funding procured	12,922,533	9,052,228	1,804,447	47,872,048
Undergraduate qualifications	98.42	83.39	0	400
Graduate qualifications	123	89.92	15	475
PhD qualifications	34.12	18.99	5	75
Material costs	1,918,139	3,855,759	691	13,684,143
Technical staff	112.92	88.9	0	400
Scientific staff	218.2	121.59	25	610
Undergraduate students	178.3	93.76	55	520
Graduate students	111.2	93.19	0	465
Citations past five years	836.4	1118.55	0	5,249
Number of observations	120			

**Table 5:** Descriptive statistics for the biology sample before imputation.

Variable	Mean	Std.Dev.	Minimum	Maximum
Publications	63.38	53.07	0	275
Third party funding procured	12,548,335	6,231,119	0	62,927,879
Undergraduate qualifications	57.98	52.79	0	260
Graduate qualifications	81.1	40.31	0	190
PhD qualifications	51.56	38.64	0	250
Material costs	979,958	1,777,236	346	10,231,855
Technical staff	65.57	34.29	0	195
Scientific staff	194.6	98.61	5	540
Undergraduate students	137.6	62.31	0	325
Graduate students	45.6	42.84	0	180
Citations past five years	1134.7	1293.11	0	7,187
Number of observations	576			

The dataset contains 696 observations of 11 variablesamounting to 7656 values of which 231 are missing, mainly regarding the third-party funding acquired and the material costs. We imputed the missing values by a random forest algorithm using the R package missForest package, which predicts the missing values in the dataset based on the observed ones in a non-parametric way This method can be applied to datasets that include both continuous and categorical variables. [42] With respect to a functional form a translog formulation has been chosen to approximate the production technology since being the second order Taylor approximation around a Cobb-Douglas function allows for the investigation of interactions between the outputs, the inputs and between the outputs and inputs. A one-sided, normally distributed error term *u* is set equal to the expression ${ln(D}_{0}\left(x,\frac{y}{y_{1}}\right))$ in (3). After adding a white noise term *v* for the statistical error to (3) delivers the following empirical specification for a latent class *c*:

$$\begin{array}{l} -\ln\left(y_{1}\right){|}_{c}=\beta_{0}+\sum_{m=2}^{M}\beta_{m}\ln\left(\frac{y_{m}}{y_{1}}\right)+\sum_{n=1}^{N}\beta_{n}\ln x_{n}+0,5\sum_{m=2}^{M}\sum_{k=1}^{M}\beta_{y_{m}y_{k}}\ln\left(\frac{y_{m}}{y_{1}}\right)\ln\left(\frac{y_{k}}{y_{1}}\right)\\ \;+0,5\sum_{n=1}^{N}\sum_{k=1}^{N}\beta_{x_{n}x_{k}}\ln x_{n}\ln x_{k}+\sum_{m=2}^{M}\sum_{n=1}^{N}\beta_{y_{m}x_{n}}\ln\left(\frac{y_{m}}{y_{1}}\right)\ln x_{n}-u+v{|}_{c} \end{array}$$ (4)

The subscripts for unit *j* and time *t* are omitted for improved readability. It should be noted that restricting all the second order interaction terms $\beta_{y_{m}y_{k}},\;\beta_{x_{n}x_{k}}$ and $\beta_{y_{m}x_{n}}$ to zero would result in the estimation of a Cobb-Douglas functional form. In order to circumvent the numerical issues in using a log-linearized model when zero values are present, we transform the dataset by adding a small number to all continuous variables following Criscuolo et al. [43], which we see as non-invasive given the rounding procedure applied to ICEland data [41]. We estimate the two models, a pooled stochastic normal frontier model and a latent class version following the work of Greene from 2005 with the Limdep 10 software [44]. We estimate a latent class model to test whether there is technological heterogeneity along sub-disciplinary lines rather than assume a priori that this is the case. The pooled stochastic frontier model is estimated in order to provide a comparison for the latent class technical efficiency estimates. A description of the latent class model from the point of view of practitioners is provided in a study by Sauer and Moreddu from 2020 [45]. Basically, the application of a latent class model results in a separation of the data into multiple technological classes [45]. This separation is achieved via the estimated probabilities of class membership, which are based on multiple pre-specified criteria summarized in a class identification vector [45]. In more detail, the latent class model estimates a multi-nomial logit model for classification of the observations together with the technological structure, which is itself estimated via weighted maximum likelihood [45]. The number of classes is determined by a “testing down” procedure as outlined in Greene [44]. The approach relies on determining the suspected number of latent classes and estimating a model with one additional latent class. The model is reestimated by stepwise reducing the number of classes by one at a time. The optimal number of classes is chosen by comparing the values of a criterium quantifying the information loss like the Akaike information criterion. A typical drawback of latent class models is posed by the fact that the theoretical distribution of the residuals in not known a priori, which makes model testing more difficult. We address this challenge by using bootstrapping methods.

The class identification vector contains the binary variable signifying the belonging to a sub-discipline, the number of PhD qualifications and citations. The number of PhD qualifications and the number of citations would be potentially useful to separate between the agriculture and biology as the descriptive statistics in Tables 4 and 5 indicate. The latent class model we estimate would be thus capable of confirming the classification into subject and research areas, should the clustering be meaningful in production technological terms.

## Estimation results and discussion

### Model selection and testing

The estimation of the translog production technology formulation in (4) for the pooled sample results in positively skewed ordinary least squares residuals. In a production frontier model we would expect a negative skew of the residuals due to the compound error [46]. A deviation from this negative skew would mean that all decision-making units are fully efficient, which is hardly plausible, but could be explained with the number of coefficients we estimate in relation to the sample size.

The two options remaining at this point are increasing the sample size to construct a sample that delivers theoretically consistent negatively skewed ordinary least squares residuals and reformulating the model [46]. The sample size in this study is fixed due to the limited number of decision-making units that fit the criteria. We chose a model reformulation and estimate the inflexible functional form the translog approximates and the corresponding latent class model. The estimation code is displayed in the Appendix II, while the estimation results are displayed in Tables 6 and 7.

**Table 6:** Estimation results for the stochastic frontier Cobb-Douglas case.

Coefficient	Estimate	Standard error	p-Value
*β*_0_ CONSTANT	.80792***	.13496	.0000
*β*_1_ GQ	.29173***	.01833	.0000
*β*_2_ UQ	.16071***	.00950	.0000
*β*_3_ F	.03521***	.00950	.0002
*β*_4_ PHDQ	.25943***	.01787	.0000
*β*_5_ TS	-.02328	.01469	.1129
*β*_6_ ScS	-.40007***	.03418	.0000
*β*_7_ GS	-.18291***	.01079	.0000
*β*_8_ US	-.19106***	.02053	.0000
*β*_9_ Cit	-.17566***	.01150	.0000
*β*_10_ MC	-.00805	.00507	.1124
Lambda	.88158***	.10031	.0000
Sigma	.32804***	.00039	.0000

Note:

***, **, * = = > Significance at 1%, 5%, 10% level. Source: own illustration.

**Table 7:** Estimation results for the stochastic frontier latent class case.

Coefficient	Estimate	Standard error	p-Value
Model parameters for latent class 1			
*β*_0_ CONSTANT	-4.09116***	.27575	.0000
*β*_1_ GQ	.32180***	.01939	.0000
*β*_2_ UQ	.05962***	.00818	.0000
*β*_3_ F	.44470***	.02164	.0000
*β*_4_ PHDQ	.07331***	.01612	.0000
*β*_5_ TS	-.04081***	.01060	.0001
*β*_6_ ScS	-.68453***	.03203	.0000
*β*_7_ GS	-.07829***	.00999	.0000
*β*_8_ US	-.11081***	.02746	.0001
*β*_9_ Cit	-.10096***	.00957	.0000
*β*_10_ MC	-.00633	.00391	.1054
Lambda	.20192***	.02513	.0000
Sigma	.79484*	.46325	.0862
Coefficient	Estimate	Standard error	p-Value
Model parameters for latent class 2			
*β*_0_ CONSTANT	1.30333***	.15459	.0000
*β*_1_ GQ	.16276***	.01982	.0000
*β*_2_ UQ	.22268***	.01551	.0000
*β*_3_ F	.00453	.00797	.5701
*β*_4_ PHDQ	.31712***	.02658	.0000
*β*_5_ TS	-.03589	.05032	.4756
*β*_6_ ScS	-.28297***	.07181	.0001
*β*_7_ GS	-.25930***	.01716	.0000
*β*_8_ US	-.16827***	.02568	.0000
*β*_9_ Cit	-.20424***	.01863	.0000
*β*_10_ MC	-.01317*	.00783	.0926
Lambda	.43215***	.02884	.0000
Sigma	3.96674***	.93897	.0000
Parameter estimates for class separating variables			
CONSTANT_CLASS_1	-2.41811	1.55811	.1207
DUMMY_CLASS_1	1.42206	1.28241	.2675
Cit_CLASS_1	.52433**	.26279	.0460
PHDQ_CLASS_1	.72284*	.42323	.0877

Note:

***, **, * = = > Significance at 1%, 5%, 10% level. Source: own illustration.

The dependent variable in both estimations is the negative logarithm of the publication count. The log-likelihood function of the stochastic frontier model was optimized at a value of -97.57293. We would fail to reject the null hypothesis of no inefficiency at common significance levels based on the *χ*^2^-distributed likelihood ratio test. The value of the Akaike information criterion associated with the stochastic frontier is 221.1.

**Fig 1:**
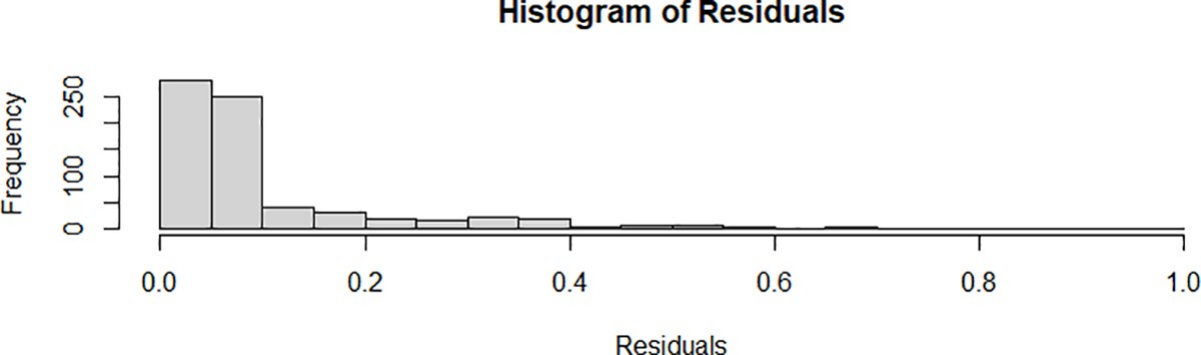
Histogram of the residuals of the latent class model with two classes. Source: own illustration.

The value of the Akaike information criterion of the latent class model with two classes is -161.9, which is considerably lower than the 221.1 obtained by the pooled model. We tested for the appropriate number of classes following the approach recommended by Greene [44]. No convergence was achieved with three latent classes, which may be explained by an overspecification of the model. We thus prefer a latent class model with two classes. Fig 1 shows a histogram of the regression residuals of the estimated latent class model with two classes.

The right skew in the residuals is to be expected by construction due to the compound error term of the two-sided, normally distributed statistical noise and the one-sided, non-negative technical inefficiency term assumed in (4). The exact distribution of the residuals is unclear, which makes the examination of model fit less than straight forward. We use bootstrapping methods to approximate the distribution of residuals empirically and hereby consider only other latent class models with two classes, which result in the same class attribution of the individual observations and instances of a normal exit from optimization as we have verified a posteriori.

We use the distribution of the residuals *R* obtained by bootstrapping to test the hypothesis of the residuals being consistent in terms of mean with their unclear theoretical distribution by constructing the following statistic *s* similar to a t-test:

$$s=\frac{R-E\left[R\right]}{Var\left(R\right)}$$ (5)

The variance of the residuals Var(R) is calculated as:

$$\mathrm{Var}\left(R\right)=E\left[R^{2}\right]-E{\left[R\right]}^{2}$$ (6)

The expected values for the residuals and the squared residuals are approximated by the sample averages in the bootstrapped sample. The estimated mean and variance for the residuals vary between the classes. Class one has an expected residual of 0.05037924 and an estimated variance of 0.00002467291, class two—an expected residual of 0.2238057 and a residual variance of 0.0000723567.

The figures in Appendix III illustrate the empirical distribution of the test statistic s for the two classes after 337 draws (S1 and S2 Figs in S3 Appendix). The values of the test statistic for latent class one cumulate around zero with some extremely low values, while the values are more evenly distributed around the median of the distribution for latent class two. We used the R package EnvStats to calculate the quantiles of its empirical distribution of the test statistic for each residual, which are then used to construct confidence intervals for the statistic.

The constructed quantiles confirm that dense distribution of the statistic in the sense that the realizations of the test statistic often overlap with the critical value. In these cases, we can reach no definite conclusion even after exhausting the 22-digit precision of the R software when comparing the values, which outlines the limitations of the R software in similar endeavors. In general, we fail to reject the null hypothesis when a conclusion can be reached. For all 696 observations we do not observe an instance of clearly rejecting the null hypothesis. This speaks in favor of the validity of the latent class regression model.

The value of the *χ*^2^ distributed likelihood ratio test associated with the latent class model also speaks in favor of the validity of the latent class model. At a test statistic value of 221.88492 with 21 degrees of freedom and a common significance level we reject the null hypothesis of all coefficients being jointly equal to zero. The prior and posterior class probabilities at data means for the latent class model variables are also consistent with a classification that improves the model fit. The average prior class probabilities are 0.72556 and 0.27444 for classes one and two respectively, while their posterior equivalents amount to 0.9809 and 0.9951.

The parameter sigma in the stochastic frontier and the latent class models informs about the proportion of variance attributed to the inefficiency term compared to the variance observed in the model. As the highly significant estimates indicate Inefficiency contributes to the overall variance to a much larger extend in the second latent class than in the first latent class. The parameter lambda gives the signal-to-noise ratio. The estimate of lambda for the first latent class is only significant at 10%, while the highly significant estimate for the second latent class indicates that there is roughly four times more signal or meaningful input than noise in the model.

### Interpretation of the estimation results

Table 7 displays the estimation results for the latent class stochastic frontier model with two classes. The first latent class has a higher median technical efficiency than the second latent class. The estimates for the deterministic part of the frontier displayed in Table 7 have the expected negative sign for the input elasticities for both latent classes. Both latent classes exhibit slightly decreasing returns to scale. The estimated input elasticities for the two latent classes outline a different production technology. A marginal increase in the production factor scientific staff would increase the aggregate output (holding the output mix constant) by approximately 0.68 percent in latent class one. The individual input contributions towards an increase in aggregate output in latent class two seem balanced in comparison. Scientific reputation reflected in the number of citations plays a more significant role in the production process of latent class two than in the latent class one. The pattern of output elasticities also diverges between the two classes.

The parameter sigma in the stochastic frontier and the latent class models informs about the proportion of variance attributed to the inefficiency term compared to the variance observed in the model. As the highly significant estimates indicate Inefficiency contributes to the overall variance to a much larger extend in the second latent class than in the first latent class. The parameter lambda gives the signal-to-noise ratio. The estimate of lambda for the first latent class is only significant at 10%, while the highly significant estimate for the second latent class indicates that there is roughly four times more signal or meaningful input than noise in the model.

The separating variables for citation counts and PhD qualifications are statistically significant for the technological latent class one at a significance level of 5 and 10% respectively. The signs of the estimated parameter indicates that increasing the number of citation counts and PhD qualifications ceteris paribus increases the likelihood of a unit being assigned to the technological latent class one. The binary variable that shows the belonging to a sub-discipline of life sciences is not significant at 10% indicating that the units are assigned to a technological latent class based on different criteria than being associated with agricultural sciences or biology. In other words: belonging to a specific sub-discipline of life sciences and the number of PhD qualifications have no bearing on a unit being assigned to a technological latent class, while the citation counts do at the 5% significance level.

**Fig 2:**
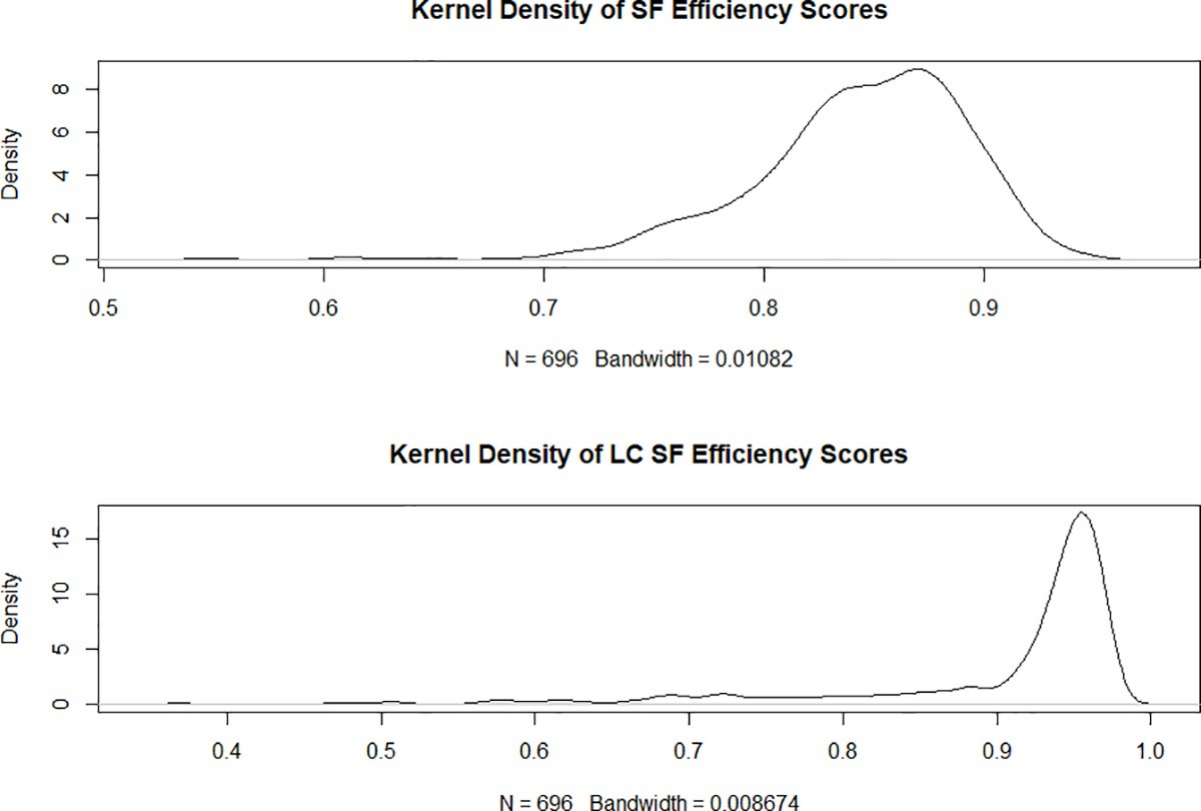
Kernel density estimate for the stochastic frontier and latent class efficiency scores for the complete sample (stochastic frontier efficiency scores above). Source: own illustration.

Having established the superior fit of the latent class model with two classes compared to the stochastic frontier model following the “testing down” procedure of Greene [44] we also observe that the stochastic frontier model seems to misinterpret heterogeneity as inefficiency. Fig 2 displays the nonparametric kernel density estimate of the technical efficiency score distribution for the stochastic frontier and latent class models The bandwidth is similar between the two density estimates, which makes their appearance comparable, since this parameter crucially influences the appearance of the estimates [47]. Both figures uncover an approximately bell-shaped form, yet the density of the technical efficiency scores associated with the latent class model is steeper and more condensed around the higher median value.

**Fig 3:**
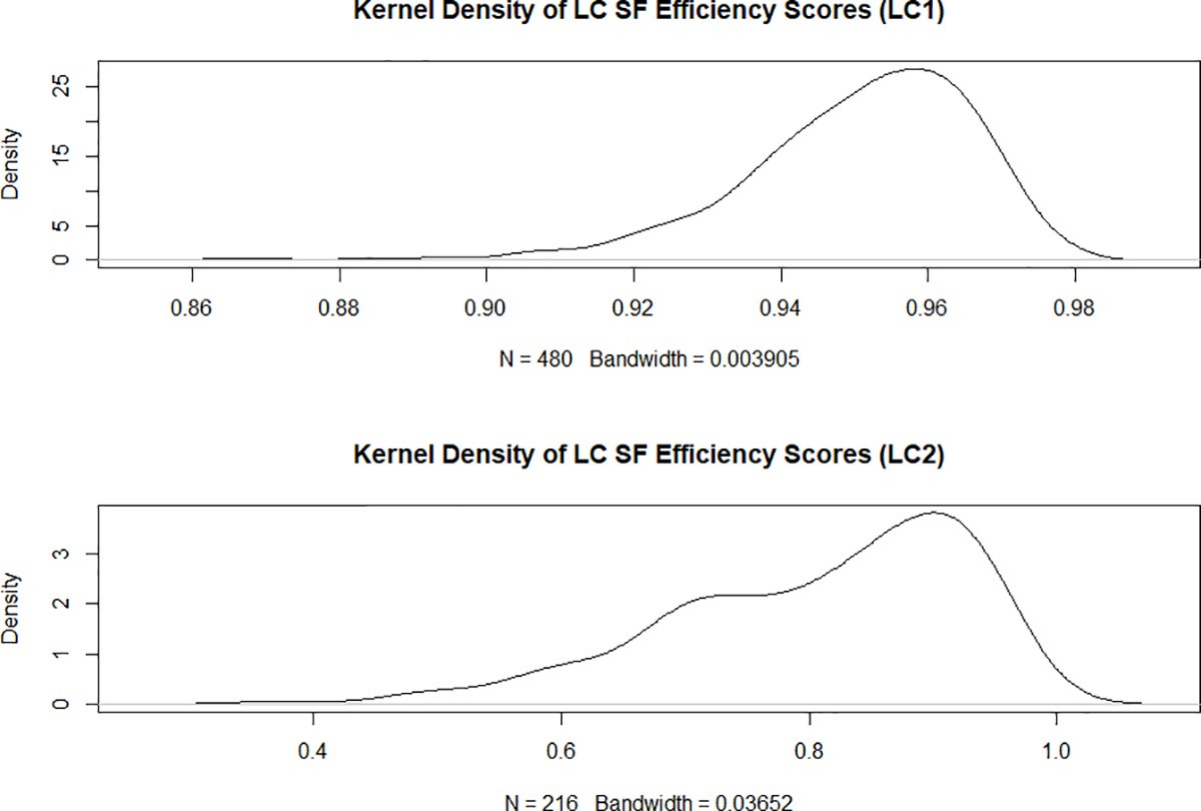
Kernel density estimate for the latent class stochastic frontier efficiency scores for the latent classes (latent class one above). Source: own illustration.

**Fig 4:**
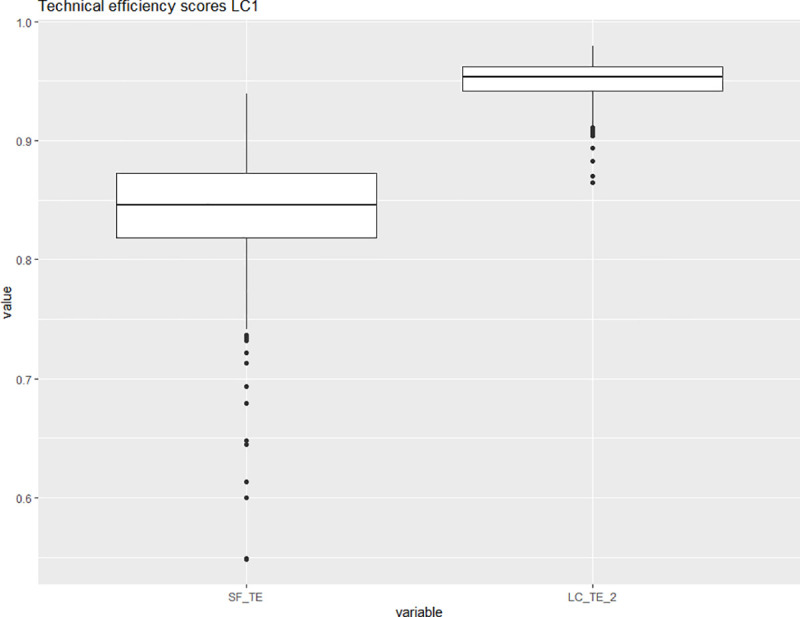
Distributions of the technical efficiency scores for the stochastic frontier (left) and the latent class model based only on the observations belonging to latent class one. Source: Own illustration.

**Fig 5:**
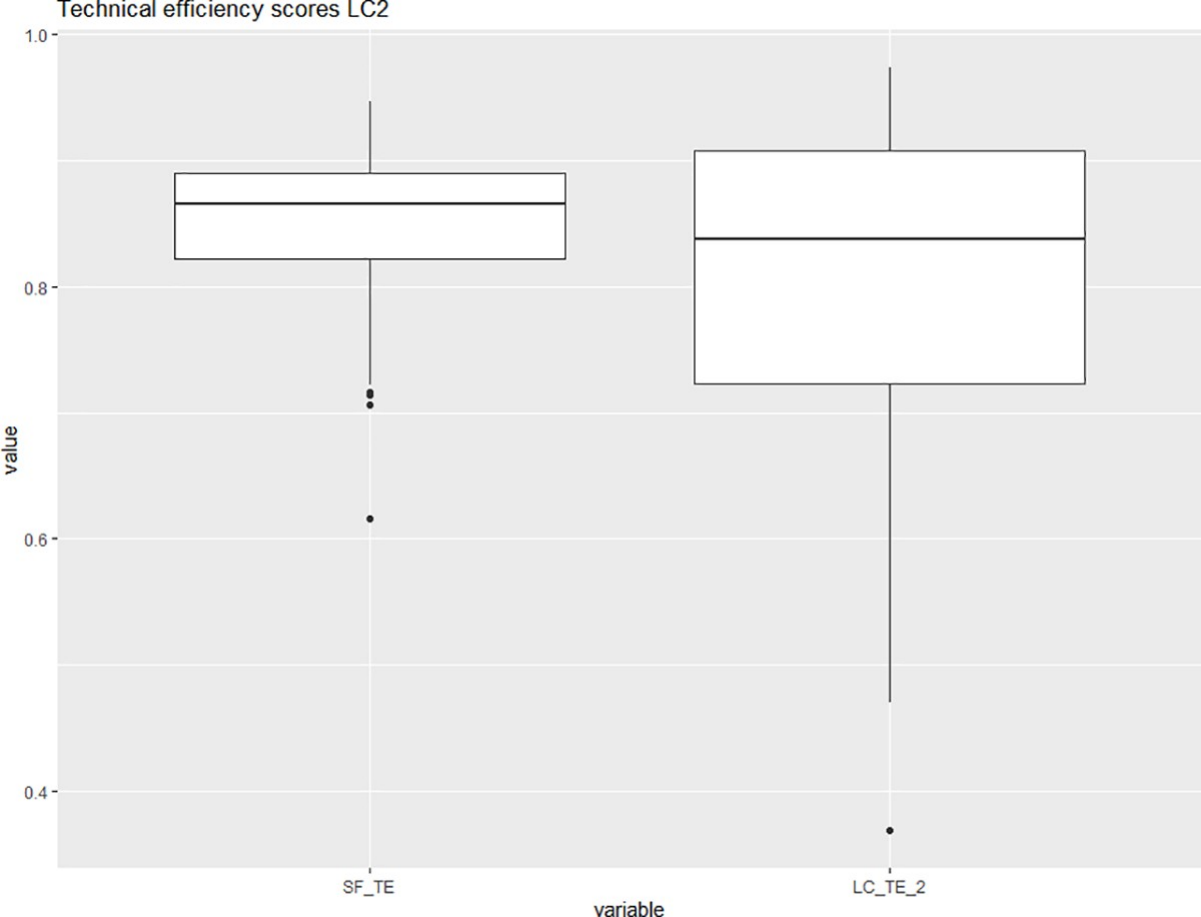
Distributions of the technical efficiency scores for the stochastic frontier (left) and the latent class model based only on the observations belonging to latent class two. Source: Own illustration.

Fig 3 shows the corresponding kernel density estimates for the two latent classes While the shape of the density estimates in Fig 3 is not directly comparable to the shape of the density estimates in Fig 2 due to the different bandwidth we observe that latent class one seems to have a more compact density with a higher median value than latent class two. This observation is reiterated in Figs 4 and 5, which via boxplots compare the distributions of the technical efficiency scores between the stochastic frontier and latent class models for the two classes separately.

Fig 4 shows that latent class one consists almost exclusively of highly efficient universities as indicated by the high median and the low dispersion of the distribution. The median technical efficiency in the latent class model is higher than the equivalent for the same observations in the pooled model. The reverse is true for latent class two as Fig 5 shows.

While the technical efficiency scores for one of the latent classes are very high similar results have been obtained in other studies utilizing stochastic frontier analysis [48]. In the context of this study this is not implausible since the latent class model effectively groups data so that the technological differences that would usually appear to be inefficiency are correctly attributed to technological heterogeneity.

**Fig 6:**
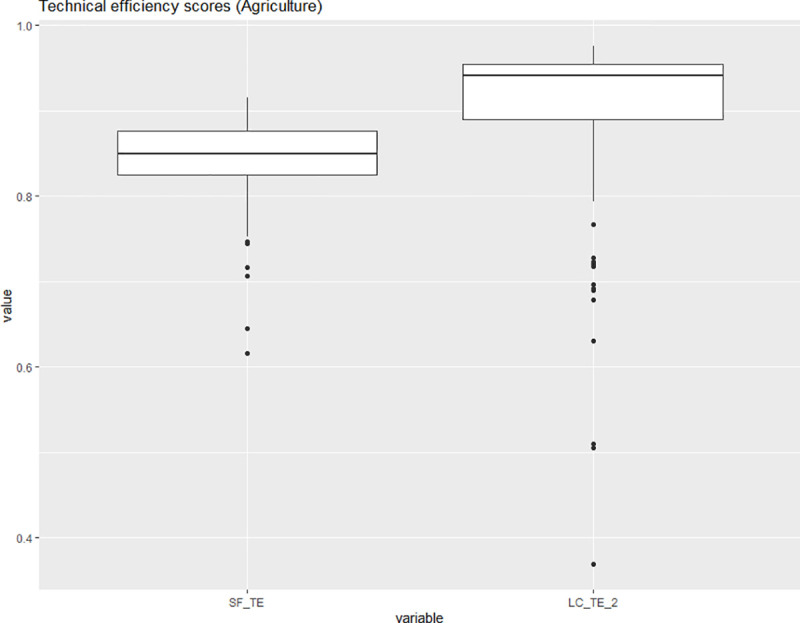
Boxplots of the technical inefficiency scores for the SFM and the LCM (agricultural sciences). Source: own illustration.

**Fig 7:**
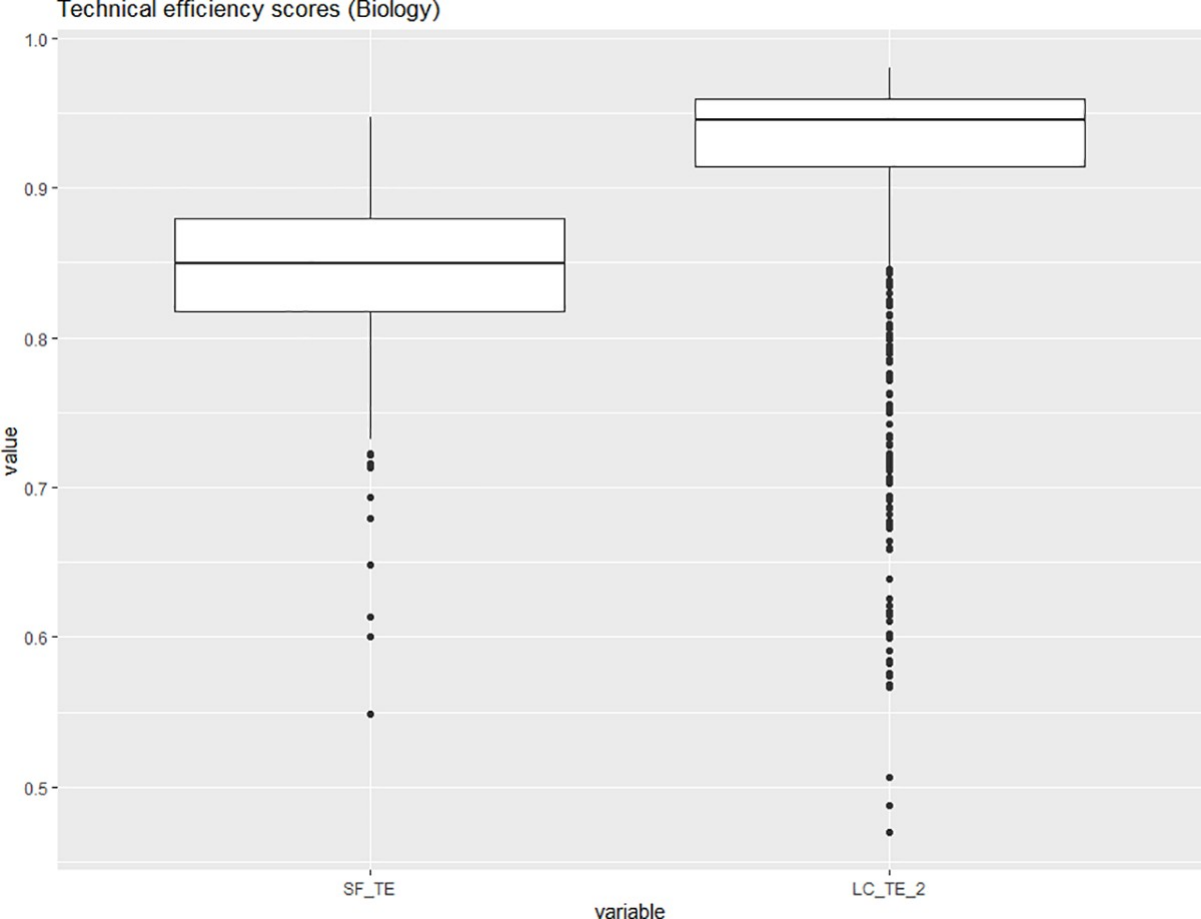
Boxplots of the technical inefficiency scores for the SFM and the LCM (biology). Source: own illustration.

Figs 6 and 7 display the distributions of the technical efficiency scores for the pooled and the latent class model based only on the observations belonging to one of the two sub-disciplines of life sciences. The classification by the latent class model raises the median estimated technical efficiency for both the agricultural sciences and the biology subsamples. The latent class model indicates that the biology subsample has a larger number of downward outliers. The mean and median technical efficiency score for the biology subsample are both approximately 0.93. Their magnitude is comparable to the mean and median technical efficiency score in the agricultural science sample, which amount to 0.93 and 0.94. This leads us to conclude that there is no systematic difference in mean technical efficiency for the agricultural and biology subsamples. The first latent class contains six of the ten agricultural science units (or 60%) and thirty-three of the forty-eight units of biology (or approximately 69%). The second latent class two contains four of the ten agricultural science units (40%) and fifteen of the forty-eight units of biology (or approximately 31%). Table 8 lists the class members for latent class two. The non-listed decision-making units are members of latent class one.

**Table 8:** Latent class two members. Source: own illustration.

Name of the university	Sub-discipline
University of Halle	Both
University of Hohenheim	Both
University of Kassel	Both
University of Rostock	Both
Technical University of Braunschweig	Biology
Technical University of Dresden	Biology
University of Bayreuth	Biology
University of Frankfurt	Biology
University of Greifswald	Biology
University of Hamburg	Biology
University of Kiel	Biology
University of Konstanz	Biology
University of Magdeburg	Biology
University of Regensburg	Biology
University of Wuppertal	Biology

**Fig 8:**
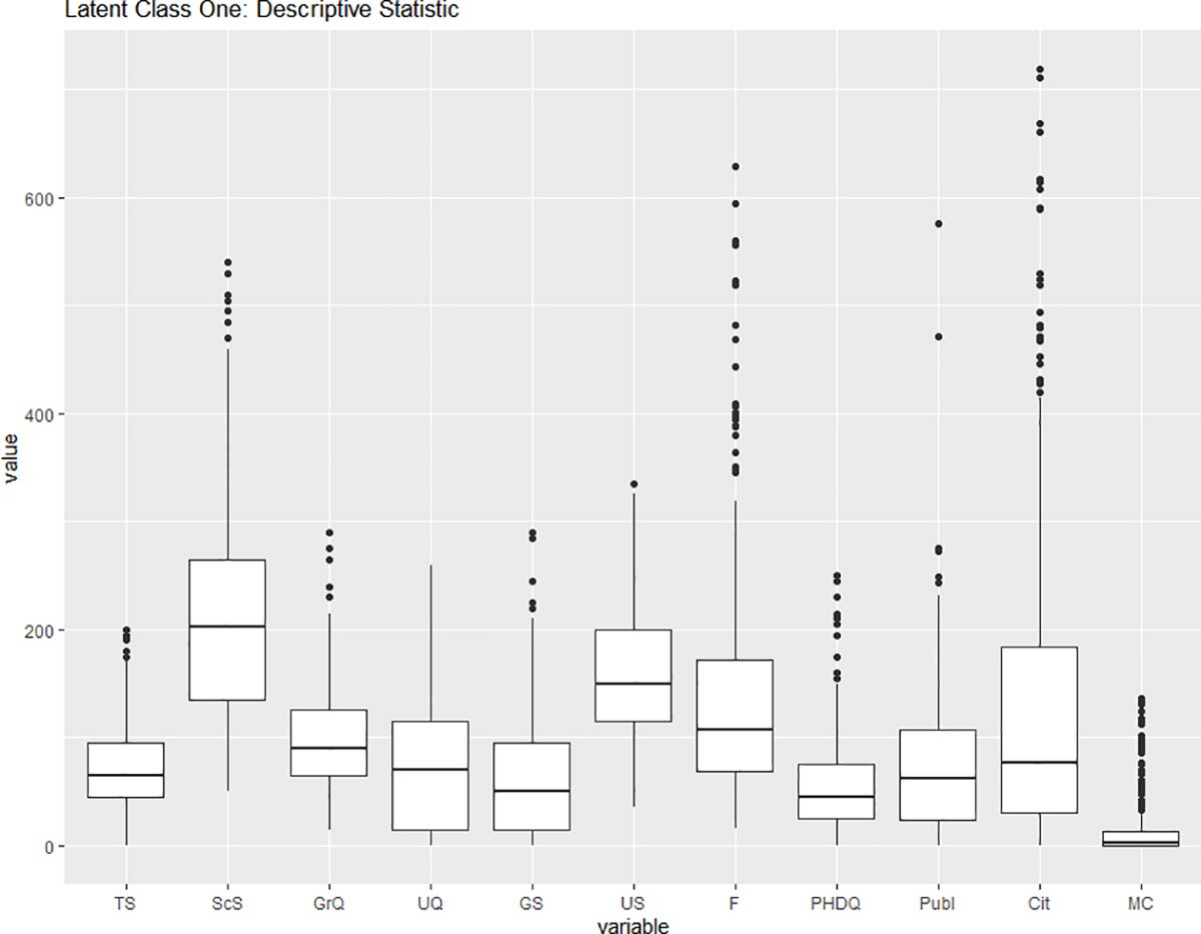
Descriptive statistic for latent class 1. Third Party Funding (F) and Material costs (MC) are displayed in 100k, while citations numbers (Cit) are displayed in tens. Source: own illustration.

**Fig 9:**
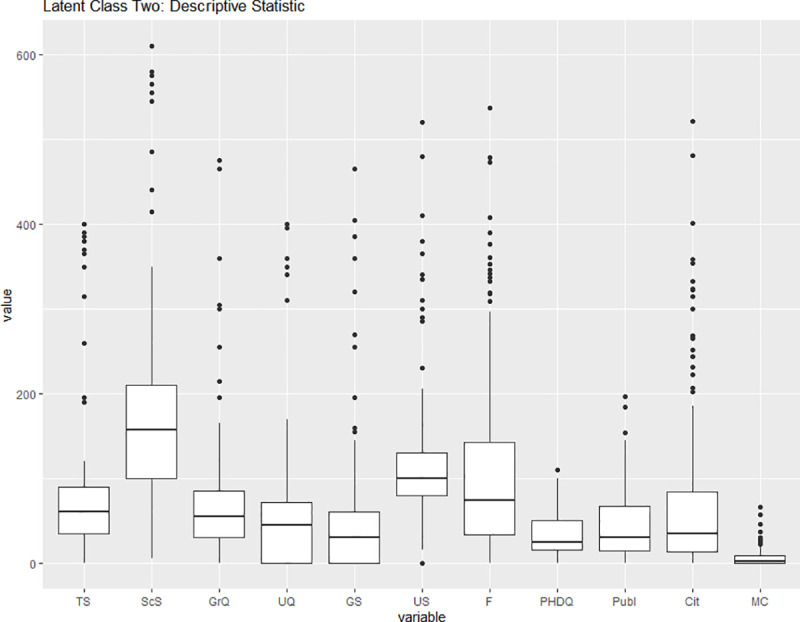
Descriptive statistic for latent class 2. Third Party Funding (F) and Material costs (MC) are displayed in 100k, while citations numbers (Cit) are displayed in tens. Source: own illustration.

While most of the biology units belong to latent class one, we assert that explaining the belonging to a latent class based on the sub-discipline of life sciences would be oversimplifying. Figs 8 and 9 display the descriptive statistic for the latent class one and the latent class two subsamples. We observe that latent class two contains the large teaching universities as can be inferred by the outliers in the numbers of undergraduate and graduate students (US and GS respectively) and undergraduate and graduate qualifications (UQ and GrQ respectively). Latent class two also exhibits lower median publication (Publ) and citation (Cit) counts. We hereby infer that the latent classes are more closely connected to the research or teaching focus of the unit than to the sub-discipline the unit belongs to. It should be noted that while some of the median values, e.g. the median of undergraduate students (US), are higher in the research-focused latent class, it is the ratio between class identification variables that allows us to interpret the latent classes.

We use a two-sample Kolmogorov-Smirnov to confirm that the distributional differences between the latent classes are statistically significant. For each of the eleven inputs and outputs we compared the two samples. The null hypothesis that the samples come from the same distribution has been rejected in all eleven cases. We report the corresponding p-value in the brackets: TS (0.04329), ScS (0.0000002824), GrQ (0.0000000000006526), UQ (0.00000005192), GS (0.0000004123), US (0.00000000000000022), F (0.000000002706), PHDQ (0.000000003482), Publ (0.00000248), Cit (0.00000003839), MC (0.005687).

## Conclusions

In this paper, we frame scientific production in a multi-output, multi-input setting to find that failing to account for the possible existence of latent classes in the data might bias the perception of efficiency in the case of the German biology and agricultural sciences. We investigate whether a latent class model would identify the different sub-disciplines of life sciences in a sample of biology and agricultural units based on technological differences. We estimate a distance function formulation via a latent class model with two classes using a unique dataset consisting of financial, personnel, examination and bibliometric data for 58 German subject and research area units. We find that allowing for heterogeneous technologies improves model fit compared to the pooled stochastic frontier model. The binary variable showing the belonging to a sub-discipline of life sciences that we used as a class separating variable is not significant, which indicates that the units are assigned to a technological latent class based on different criteria than the sub-discipline. Our results indicate that a technological separation in research and teaching-oriented classes is more meaningful that a technological separation along sub-disciplinary lines. In fact, we find no evidence of a difference between German agricultural sciences and biology in terms of mean technical efficiencies in the latent class model.

This approach accounts for heterogeneity like the contributions of Agasisti and Johnes from 2010 and Johnes and Johnes from 2016, which both conduct a university level analysis [4, 5]. Their estimates are not immediately comparable to our estimates since we have chosen a primal, not a dual formulation of the production technology. This means that we investigate the technical efficiency of units rather than the allocative efficiency. Like the studies of Gralka, Wohlrabe and Bornmann from 2019 and Bornmann, Gralka, de Moya Anegón and Wohlrabe from 2020 our work acknowledges the importance of publication counts and research grants as outputs in the case of higher education [29, 49]. Unlike these two recent studies we account for the possibility that there are unobserved latent classes in the data. Like Wohlrabe and Gralka contribution from 2020 we consider heterogeneity, but we also investigate the effects of this heterogeneity on the technical efficiency of the units [6].

With respect to further work we propose an estimation of the flexible translog model account for interactions between inputs and outputs and the theoretically consistent skewness of the ordinary least squares residuals enforced in a fashion similar to what is suggested in Sauer et al. [50]. The use of Bayesian methods could alleviate the limitations posed by the sample size as we would not have to rely on asymptotics for valid statistical inference in this case. This would allow for the estimation of the trade-offs posed by producing multiple outputs. Heterogeneity over time and the role of technical progress could also be investigated. The social capital of the researchers could also be considered. Further research could account for the third mission of universities and add controls for possible differences in the production environment, e.g. compare the situation in different countries.

In conclusion, there already exists a variety of research applying efficiency models in education and examining diverse constellations of different inputs and outputs. Still, there is much more left to explore, since not only the methodologies are evolving. The means to record and supply data are advancing and in the future panel data evaluations may become of greater importance to differentiate between static and dynamic effects. Furthermore, cross-country comparisons might increase in relevance and transparency with easier data access. Nevertheless the heterogeneity of different units should be kept in mind and instead of focusing on larger units, attention might rather be paid to analyzing smaller units and exploring whether heterogeneity posed by different focus, e.g. research or teaching, might be misinterpreted as inefficiency.

## Supporting information

S1 Appendix (DOCX)

S2 Appendix (DOCX)

S3 Appendix (DOCX)
