# Supplementary material for: How efficient are German life sciences? Econometric evidence from a latent class stochastic output distance model
Source: PLoS One. 2021 Mar 12;16(3):e0247437. doi: 10.1371/journal.pone.0247437 (PMC7954326; doi:10.1371/journal.pone.0247437)
Supplement: S1 Appendix — (DOCX) [file pone.0247437.s001.docx]

S1 Appendix.

**S1 Table. Universities in the sample. Source: own illustration.**

| Humboldt University of Berlin | University of Hamburg |
| --- | --- |
| Technical University of Munich | University of Hannover |
| University of Bonn | University of Heidelberg |
| University of Giessen | University of Jena |
| University of Goettingen | University of Cologne |
| University of Halle | University of Konstanz |
| University of Hohenheim | University of Leipzig |
| University of Kassel | University of Magdeburg |
| University of Kiel | University of Mainz |
| University of Rostock | University of Marburg |
| Free University of Berlin | University of Munich |
| Karlsruher Institute for Technology (KIT) | University of Muenster |
| Technical University Aachen | University of Oldenburg |
| Technical University of Braunschweig | University of Osnabrueck |
| Technical University of Darmstadt | University of Potsdam |
| Technical University of Dresden | University of Regensburg |
| Technical University of Kaiserslautern | University of Stutttgart |
| University of Bayreuth | University of Tuebingen |
| University of Bielefeld | University of Ulm |
| University of Bochum | University of Wueppertal |
| University of Bremen | University of Wuerzburg |
| University of des Saarlandes Saarbrücken |  |
| University of Düsseldorf |  |
| University of Erlangen Nürnberg |  |
| University of Frankfurt |  |
| University of Freiburg |  |
| University of Greifswald |  |
