# Supplementary material for: How efficient are German life sciences? Econometric evidence from a latent class stochastic output distance model
Source: PLoS One. 2021 Mar 12;16(3):e0247437. doi: 10.1371/journal.pone.0247437 (PMC7954326; doi:10.1371/journal.pone.0247437)
Supplement: S2 Appendix — (DOCX) [file pone.0247437.s002.docx]

S2 Appendix.

---------------------------------------------------------------------------

Limited Dependent Variable Model - FRONTIER

Dependent variable NEPU

Log likelihood function -97.57293

Estimation based on N = 696, K = 13

Inf.Cr.AIC = 221.1 AIC/N = .318

Model estimated: Jul 07, 2020, 20:56:21

Variances: Sigma-squared(v)= .06055

Sigma(v) = .24607

Sigma-squared(u)= .04706

Sigma(u) = .21693

Sigma = Sqr[(s^2(u)+s^2(v)]= .32804

Gamma = sigma(u)^2/sigma^2 = .43731

Var[u]/{Var[u]+Var[v]} = .22022

Stochastic Production Frontier, e = v-u

-----[ Tests vs. No Inefficiency ]-----

LR test for inefficiency vs. OLS v only

Deg. freedom for sigma-squared(u): 1

Deg. freedom for heteroscedasticity: 0

Deg. freedom for truncation mean: 0

Deg. freedom for inefficiency model: 1

LogL when sigma(u)=0 -98.31647

Chi-sq=2*[LogL(SF)-LogL(LS)] = 1.487

Kodde-Palm C*: 95%: 2.706, 99%: 5.412

LM test for sigma(u) = 0 based on ols e

Chi-sq[1]=(N/6)*[m3/s^3]^2 .140

Wald tests based on MLEs shown in table

--------+------------------------------------------------------------------

| Standard Prob. 95% Confidence

NEPU| Coefficient Error z |z|>Z* Interval

--------+------------------------------------------------------------------

|Deterministic Component of Stochastic Frontier Model

Constant| .80792*** .13496 5.99 .0000 .54341 1.07243

MSCABSCH| .29173*** .01833 15.91 .0000 .25580 .32766

BSCABSCH| .16071*** .00950 16.92 .0000 .14209 .17932

DRITTM| .03521*** .00950 3.70 .0002 .01658 .05383

PROMOTIO| .25943*** .01787 14.52 .0000 .22440 .29445

TECHNPE| -.02328 .01469 -1.59 .1129 -.05207 .00550

WISSPE| -.40007*** .03418 -11.70 .0000 -.46707 -.33307

MSCSTUDE| -.18291*** .01079 -16.95 .0000 -.20406 -.16176

BSCSTUDE| -.19106*** .02053 -9.31 .0000 -.23131 -.15082

ZITIERUN| -.17566*** .01150 -15.28 .0000 -.19819 -.15313

SACHKOST| -.00805 .00507 -1.59 .1124 -.01798 .00189

|Variance parameters for compound error

Lambda| .88158*** .10031 8.79 .0000 .68499 1.07818

Sigma| .32804*** .00039 836.82 .0000 .32727 .32880

--------+------------------------------------------------------------------

Note: ***, **, * ==> Significance at 1%, 5%, 10% level.

---------------------------------------------------------------------------

|-> FRONTIER;LHS=NePu;RHS= ONE, MSCABSCH, BSCABSCH, DRITTM, PROMOTIO, TECHNPE, WISSPE, MSCSTUDE, BSCSTUDE, ZITIERUN, SACHKOST;

LCM= DUMMY, ZITIERUN, PROMOTIO; PTS=2; Techeff = LC_TE_2; Panel; Maxit = 200; Alg = BFGS; RES= Resid; Group= class$

+-----------------------------------------------------------------+

| Variable = ____________ Variable Groups Max Min Average |

| JAHR Group sizes UNI 58 12 12 12.0 |

+-----------------------------------------------------------------+

+------------------------------------------------------+

| Frequency count for group sizes of JAHR |

| Group size = 12 Pct = 100.00% CumPct = 100.00% |

+------------------------------------------------------+

Normal exit: 76 iterations. Status=0, F= -110.9425

---------------------------------------------------------------------------

Latent Class / Panel Frontier Model

Dependent variable NEPU

Log likelihood function 110.94246

Restricted log likelihood .00000

Chi squared [ 21 d.f.] 221.88492

Significance level .00000

Estimation based on N = 696, K = 30

Inf.Cr.AIC = -161.9 AIC/N = -.233

Model estimated: Jul 07, 2020, 20:56:22

Unbalanced panel has 58 individuals

Stochastic frontier (half normal model)

--------+------------------------------------------------------------------

| Standard Prob. 95% Confidence

NEPU| Coefficient Error z |z|>Z* Interval

--------+------------------------------------------------------------------

|Model parameters for latent class 1

Constant| -4.09116*** .27575 -14.84 .0000 -4.63161 -3.55071

MSCABSCH| .32180*** .01939 16.60 .0000 .28380 .35980

BSCABSCH| .05962*** .00818 7.29 .0000 .04359 .07566

DRITTM| .44470*** .02164 20.55 .0000 .40229 .48710

PROMOTIO| .07331*** .01612 4.55 .0000 .04172 .10490

TECHNPE| -.04081*** .01060 -3.85 .0001 -.06159 -.02003

WISSPE| -.68453*** .03203 -21.37 .0000 -.74731 -.62176

MSCSTUDE| -.07829*** .00999 -7.83 .0000 -.09787 -.05870

BSCSTUDE| -.11081*** .02746 -4.04 .0001 -.16463 -.05700

ZITIERUN| -.10096*** .00957 -10.55 .0000 -.11971 -.08220

SACHKOST| -.00633 .00391 -1.62 .1054 -.01399 .00133

Sigma| .20192*** .02513 8.03 .0000 .15265 .25118

Lambda| .79484* .46325 1.72 .0862 -.11312 1.70280

|Model parameters for latent class 2

Constant| 1.30333*** .15459 8.43 .0000 1.00033 1.60633

MSCABSCH| .16276*** .01982 8.21 .0000 .12391 .20161

BSCABSCH| .22268*** .01551 14.36 .0000 .19228 .25308

DRITTM| .00453 .00797 .57 .5701 -.01110 .02015

PROMOTIO| .31712*** .02658 11.93 .0000 .26503 .36921

TECHNPE| -.03589 .05032 -.71 .4756 -.13452 .06273

WISSPE| -.28297*** .07181 -3.94 .0001 -.42372 -.14222

MSCSTUDE| -.25930*** .01716 -15.11 .0000 -.29294 -.22567

BSCSTUDE| -.16827*** .02568 -6.55 .0000 -.21859 -.11794

ZITIERUN| -.20424*** .01863 -10.96 .0000 -.24075 -.16773

SACHKOST| -.01317* .00783 -1.68 .0926 -.02852 .00218

Sigma| .43215*** .02884 14.98 .0000 .37562 .48868

Lambda| 3.96674*** .93897 4.22 .0000 2.12640 5.80709

|Estimated prior probabilities for class membership

ONE_1| -2.41811 1.55811 -1.55 .1207 -5.47195 .63574

DUMMY_1| 1.42206 1.28241 1.11 .2675 -1.09143 3.93554

ZITIER_1| .52433** .26279 2.00 .0460 .00928 1.03938

PROMOT_1| .72284* .42323 1.71 .0877 -.10668 1.55236

ONE_2| 0.0 .....(Fixed Parameter).....

DUMMY_2| 0.0 .....(Fixed Parameter).....

ZITIER_2| 0.0 .....(Fixed Parameter).....

PROMOT_2| 0.0 .....(Fixed Parameter).....

--------+------------------------------------------------------------------

Note: ***, **, * ==> Significance at 1%, 5%, 10% level.

Fixed parameter ... is constrained to equal the value or

had a nonpositive st.error because of an earlier problem.

---------------------------------------------------------------------------

---------------------------------------------------------------------------

Prior class probabilities at data means for LCM variables

Class 1 Class 2

.72556 .27444
