# Supplementary material for: How efficient are German life sciences? Econometric evidence from a latent class stochastic output distance model
Source: PLoS One. 2021 Mar 12;16(3):e0247437. doi: 10.1371/journal.pone.0247437 (PMC7954326; doi:10.1371/journal.pone.0247437)

S3 Appendix.

**S1 Fig**. **Distribution of the test statistic for class 1 (480 observations).** Source: own illustration.


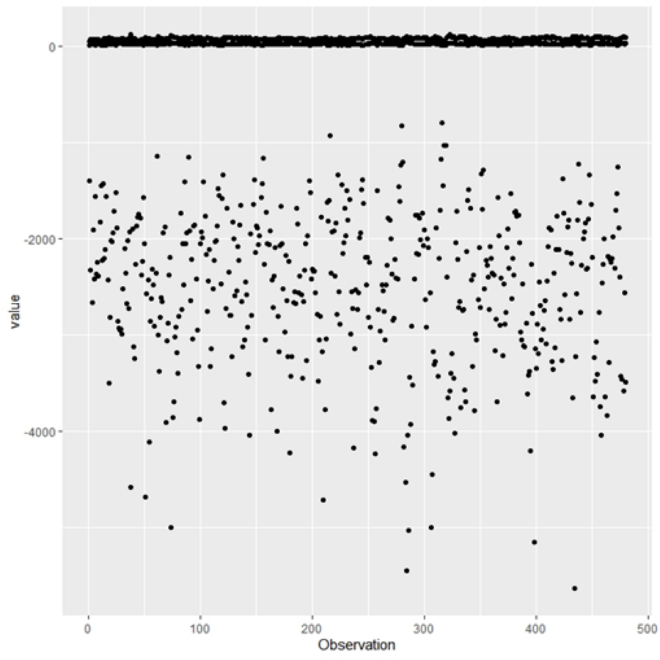


**S2 Fig.** **Distribution of the test statistic for latent class 2 (216 observations).** Source: own illustration.


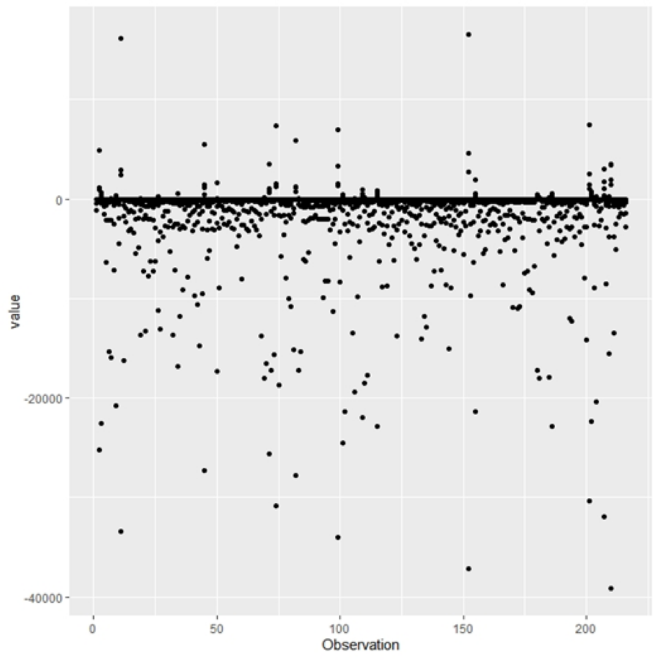

Supplement: S3 Appendix — (DOCX) [file pone.0247437.s003.docx]
